# Method to determine the decolorization potential of persistent dyes by white rot fungi by colorimetric assays

**DOI:** 10.1016/j.mex.2022.101885

**Published:** 2022-10-22

**Authors:** Christian Zafiu, Seta Küpcü, Mika A. Kähkönen

**Affiliations:** a University of Natural Resources and Life Sciences, Vienna, Department of Water-Atmosphere-Environment, Institute of Waste Management and Circularity, Muthgasse 107, Vienna 1190, Austria; b University of Natural Resources and Life Sciences, Vienna, Department of Nanobiotechnology, Institute for Synthetic Bioarchitectures, Muthgasse 11, Vienna 1190, Austria; c Department of Microbiology (Biocenter 1, Viikinkaari 9), Faculty of Agriculture and Forestry, University of Helsinki, Finland

**Keywords:** Textile dyes, White rot fungi, Degradation, Colorimetric assay, Decolorization, A_0_, Absorbance at sampling point 0 days, At, Absorbance at sampling point t days, k, rate constant, R², coefficient of determination, RB5, textile dye “Reactive Black 5”, RB4, textile dye “Reactive Blue 4”, RG19, textile dye “Reactive Green 19”, RO16, textile dye “Reactive Orange 16”, t, time

## Abstract

Decolorization assays allow to assess the ability of white rot fungi to degrade persistent organic molecules such as textile dyes and can contribute to discover microorganisms that can be used for bioremediation. The decolorization can be overlayed by the absorption from metabolites that are produced by fungi during screening, which interfere with the results. To compensate for this interference a method was developed by using different controls to subtract interfering signals.

The method was designed for simple screening in multiwell plates that can be operated with a plate reader. It was applied to four different textile dyes (Reactive Black 5, Reactive Blue 4, Reactive Green 19, and Reactive Orange 16) that were degraded by the white rot fungus *Phanerochaete velutina*. The four textile dyes showed different results with a different degree of interference. The controls allow to compensate for interfering signals and to calculate kinetic parameters for the decolorization reaction and the enzymatic degradation.

• Determine the non-enzymatic degradation of the dyes in experiments without fungi.
• Determine the absorbance of metabolites and subtract it from the decolorization data to obtain the degradation of the dye.
• Determine kinetic parameters of the degradation to compare the efficiency of the enzymes towards dyes.

## Graphical abstract

**Figure:**
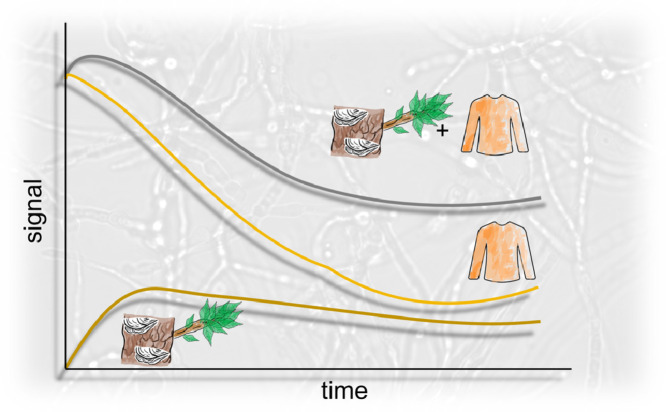

Specifications table

**Table:** 

Subject area:	Environmental Science
More specific subject area:	Decolorization potential of fungi
Name of your method:	Subtraction of background signals intrinsically produced by white rot fungi in colorimetric assays.
Name and reference of original method:	C. Zafiu, F. Part, E.-K. Ehmoser, M.A. Kähkönen, Investigations on inhibitory effects of nickel and cobalt salts on the decolorization of textile dyes by the white rot fungus *Phanerochaete velutina*, Ecotoxicology and Environmental Safety 215 (2021) 112093. https://doi.org/10.1016/j.ecoenv.2021.112093.
Resource availability:	N.A.

**Method details**

## Introduction

Screening technologies can help to improve the application of white rot fungi for bioremediation strategies, exploring the efficiency of the enzymatic mixes of individual species, and selecting such promising species for the extraction of their enzymes for further biotechnological improvements and production. Colorimetric assays in combinations with xenobiotic reporters, such as textile dyes, could be used in future for such purposes, since they offer a rapid, simple, and cheap screening procedure. However, since the underlying biological processes are complex in microorganisms and often not fully understood, it is necessary to explore these types of assays in depth. An earlier study showed that colorimetric assays that were used to explore the decolorization of textile dyes was suitable to quantify the degradation of Reactive Orange 16 (RO16), but could not be used for Remazol Brilliant Blue R (RBBR), due to an unexpected increase in absorbance, which was attributed to a metabolite of the fungus [1]. An open question was to understand to which extent the absorbance, that was used to observe the decolorization of RO-16, was influenced by metabolites of the fungus and alters the outcomes of such assays. Therefore, the method for screening of white rot fungi in colorimetric assay was studied and the procedure and outcomes thoroughly discussed for RO16 and on 3 additional textile dyes (Reactive Black 5, Reactive Blue 4, and Reactive Green 19) that are frequently used in the textile industry.

## Method

This method was established by using *Phanerochaete velutina* (FBCC 941, previous number 244i) and can be applied to other white rot fungi. We present here the results of four persistent textile dyes. However, the method can be applied to on any dye and any white rot fungus. The investigated dyes were Reactive Orange 16 (Sigma-Aldrich, Germany, CAS, no. 12225-83-1, referred as “RO16”), Reactive Black 5 (Sigma-Aldrich, Germany, CAS, no. 17095-24-8, referred as “RB5”), Reactive Blue 4 (Sigma-Aldrich, Germany, CAS, no. 13324-20-4, referred as “RB4”), and Reactive Green 19 (Sigma-Aldrich, Germany, CAS, no. 61931-49-5, referred as “RG19”).

We present and discuss the method step by step providing methodological steps that are necessary to obtain kinetic data on the overall decolorization, and the decolorization caused by non-enzymatic degradation, as well as the decolorization caused by enzymatic degradation.

### Test cultivations

*P. velutina* (FBCC 941, previous number 244i) was obtained from the Fungal Biotechnology Culture Collection (FBCC) at the Department of Microbiology at the University of Helsinki in Finland.

• *P. velutina* was pre-grown on malt extract-agar (MEA) plates during seven days at 28°C.
• Five MEA plugs (4 mm diameter) of pre-grown fungus were added to sterilized LN-AS medium (pH = 4.5; 75 ml).
• The media had finally 0.5 % (wt/ vol) glucose as carbon source.
• The liquid medium for pre-growth incubations were made in two 250 ml flasks, which were shaken during seven days at 28°C.
• Both pre-growth liquid cultures were combined and mixed with a sterile stirrer.

### Assay

Four different plates were prepared with samples that contained different textile dyes (D) and the same fungus (F).

For setting up this type of assay at least four different experiments must be prepared, which should be investigated at the same time as the main decolorization experiment.

• Growth medium without dyes and without fungus to obtain the background absorption signal of the matrix. (-D -F)
• Growth medium with dyes and without fungus to obtain the oxidative decolorization of the dye in absence of the fungus. (+D -F)
• Growth medium without dye and with fungus to obtain the changes in absorbance that are induced by the fungus only. (-D +F)
• Growth medium with dyes and fungus to obtain the absorbance change of the colorization. (+D +F)

If the dye is already known to be persistent under the experimental conditions in absence of microorganisms, the experiments +D -F may be omitted.

In case of experiments that use the decolorization reaction as an observable for the vitality of the fungus under the influence of (potential) toxins, additional experiments should be planned to investigate the influence of the toxin towards enhancing oxidative degradation of the dye and/or adding to the background absorption.

The experiments were prepared in transparent 48-wellplates that were compatible with a microtiter reader (Tecan Infinite 200 plate reader, Austria). Stock solutions of dyes with a concentration of 500 mg l^−1^ in made in sterilized growth medium. Each experiment was prepared by adding first MilliQ water, then growth medium, then dye stock solution, and finally fungus stock solution. Each experiment was conducted in quadruplicates. The multiwellplates were sealed after all solutions were added. Between the sampling points the multiwellplates were kept at 28°C.

The wavelength with the strongest absorption was chosen from the experiment without fungus and using the dye only (+D -F) at the initial timepoint (t=0) when the experiment started. The wavelengths with the strongest absorption were 502 nm for RO16, 597 nm for RB5, 595 nm for RB4, and 630 nm for RG19.

In the present case measurements were taken at 0, 6, 9, 14, and 18 days in case of experiments with RB5, RB4, and RG19, while RO16 was measured after 0, 3, 8, 14 and 19 days, as RO16 was investigated in independent experiments.

### Assay results and calculations

**Fig. 1:**
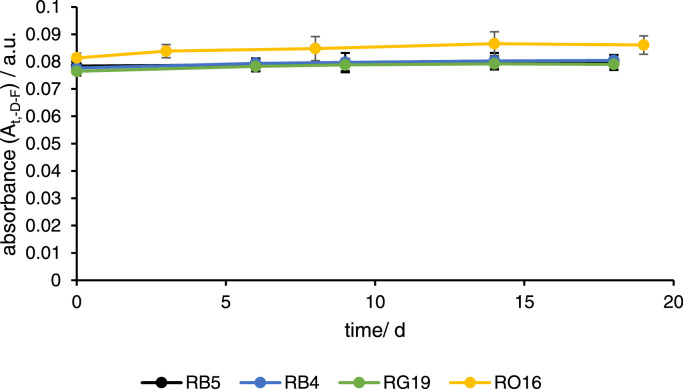
Absorption transients of background measurements of solutions without dyes and without fungi (-D -F) measured at different wavelengths with 597 nm for RB5 (black), 595 nm for RB4 (blue), 630 nm for RG19 (green), and 502 nm for RO16 (dark yellow). The absorbance is shown as mean of quadruplicates and the error bars represent the standard deviation.

The background measurements were made in growth medium without dyes and without fungi (-D -F) and showed the changes in background absorbance over time (Fig. 1), which was slightly higher at 504 nm with a mean value overall sampling points of 0.084±1.9 × 10^−3^ a.u. (absorption units) than at 595 nm (0.079±7 × 10^−4^ a.u.), 597 nm (0.079±1.0 × 10^−3^ a.u), and 630 nm (0.78±1.0 × 10^−3^ a.u). The background was quite stable, which was calculated as linear slope with a change of 1.0 × 10^−^^4^ a.u. d^−1^ at 597 nm, 1.5 × 10^−^^4^ a.u. d^−1^ at 595 nm, 1.4 × 10^−^^4^ a.u. d^−1^ at 630 nm, and 2.4 × 10^−^^4^ a.u. d^−1^ at 502 nm, which means that in the worst case (502 nm), the absorbance change from the background was 0.0045 a.u. over the whole experiment (19 days).

**Fig. 2:**
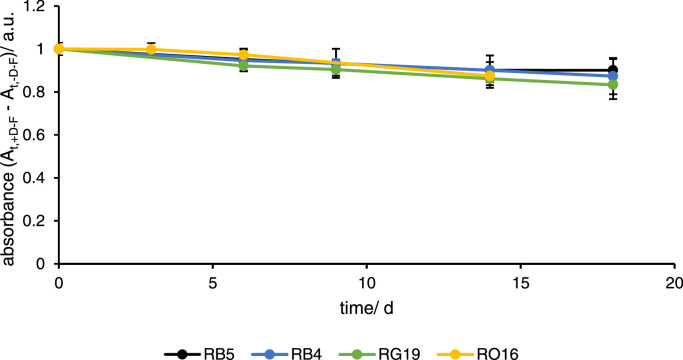
Absorption transients of measurements of solutions with dyes and without fungi (+D -F) measured at different wavelengths with 597 nm for RB5 (black), 595 nm for RB4 (blue), 630 nm for RG19 (green), and 502 nm for RO16 (dark yellow). Each absorbance value was subtracted from the absorbance of the background (-D-F) at each sampling point. The absorbance is shown as mean of quadruplicates and the error bars represent the standard deviation.

The dyes exhibited different absorbances at the initial sampling points, although they had almost the same concentration, due to their absorptivity (Fig. 2). The absorbance of “+D-F” was subtracted by the mean absorbance of “-D-F” at each sampling point to obtain the pure decline of absorbance without influence of the background absorption.

The most intense dye was RO16, that reached 0.950 a.u., followed by RB5 with 0.383 a.u., RG19 0.250 a.u., and RB4 with 0.117 a.u. All dyes showed a non-enzymatic decolorization, which was calculated by a linear model with high linearity, indicated by coefficients of determination (R²) >0.9. Non-enzymatic decolourisation was fastest for RO16 with -0.001 a.u. d^−1^ (R²=0.968), followed by RG19 (R²=0.976), RB5 with -0.002 a.u. d^−1^ ^(R²=0.937)^, and RB4 with -0.0008 a.u. d^−1^ (R²=0.992). In the worst case of RO16, this would mean that a reduction of up to 0.18 a.u. was possible within 19 days, without enzymes from the fungus. For the other dyes, a much lower reduction of 0.014 – 0.040 a.u. was found within 18 days.

**Fig. 3:**
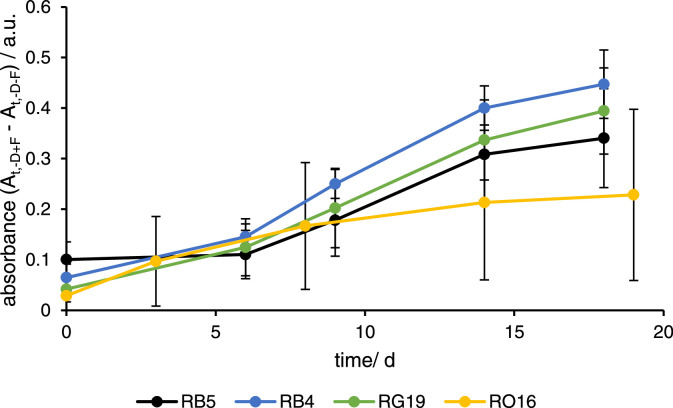
Absorption transients of measurements of solutions with dyes and without fungi (+D -F) measured at different wavelengths with 597 nm for RB5 (black), 595 nm for RB4 (blue), 630 nm for RG19 (green), and 502 nm for RO16 (dark yellow). Each absorbance value was subtracted by the absorbance of the background (-D-F) at each sampling point. The absorbance is shown as mean of quadruplicates and the error bars represent the standard deviation.

Experiments that contain only fungi and no dyes are recommended, as it turned out in an earlier study [1], that unexpected absorbance increases can be found in colorimetric assays. These interfering absorbance increase originated most likely from metabolites of the fungi. Therefore, experiments should be conducted, in which no dyes are added and only the change of the background absorption caused by the fungi at the wavelength of interest in growth medium is analysed (Fig. 3). The transients show that an absorption increase was found at each investigated wavelength, which must be considered during decolorization experiments. The transients appear non-linear and have a sigmoidal shape that saturated after 18 days, and might have resulted from a reduced metabolism and proliferation of the fungi caused by the lack of nutrients after 18 days. In addition, the absorbance grew stronger at higher wavelengths than at lower ones following the order: 595>630>597>>502 nm.

(1) $$\Delta A_{t,\left(+D-F\right)}=A_{0,\left(+D-F\right)}-A_{t,\left(+D-F\right)}$$

Eq. 1: Calculation of the contribution of the non-enzymatic degradation of dyes.

**Fig. 4:**
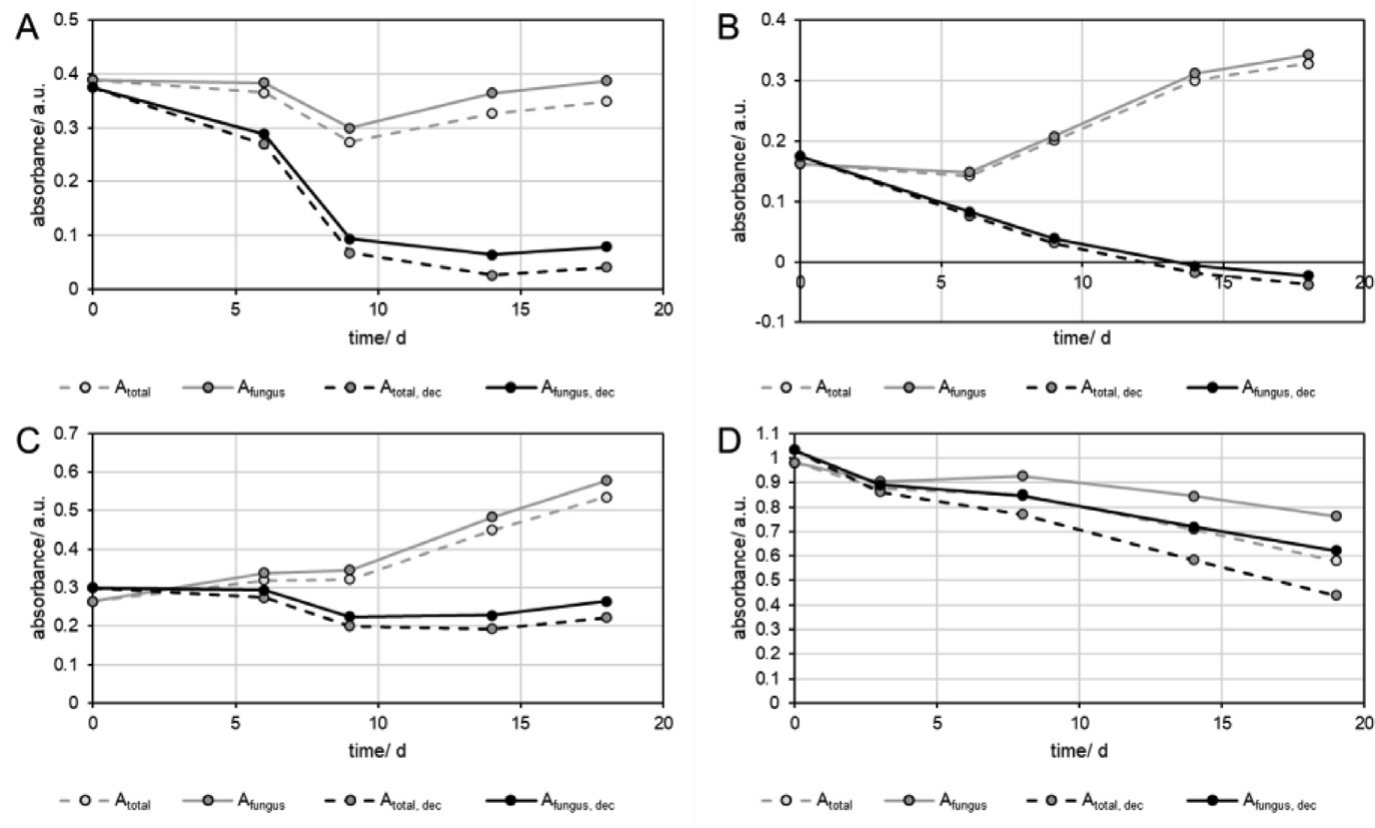
Absorption transients of measurements of solutions with dyes and with fungi (+D +F) obtained at different wavelengths with A) 597 nm for RB5, B) 595 nm for RB4, C) 630 nm for RG19, and D) 502 nm for RO16. The absorbance values were subtracted from the absorbance of the background (A_total_; grey dashed),from the samples that contained only dye and no fungus (A_fungus_), from A_total_ (A_total, dec_, black dashed), and from A_total,dec_ (A_fungus, dec_, black solid), at each sampling point. The absorbance is shown as mean of quadruplicates.

**Fig. 5:**
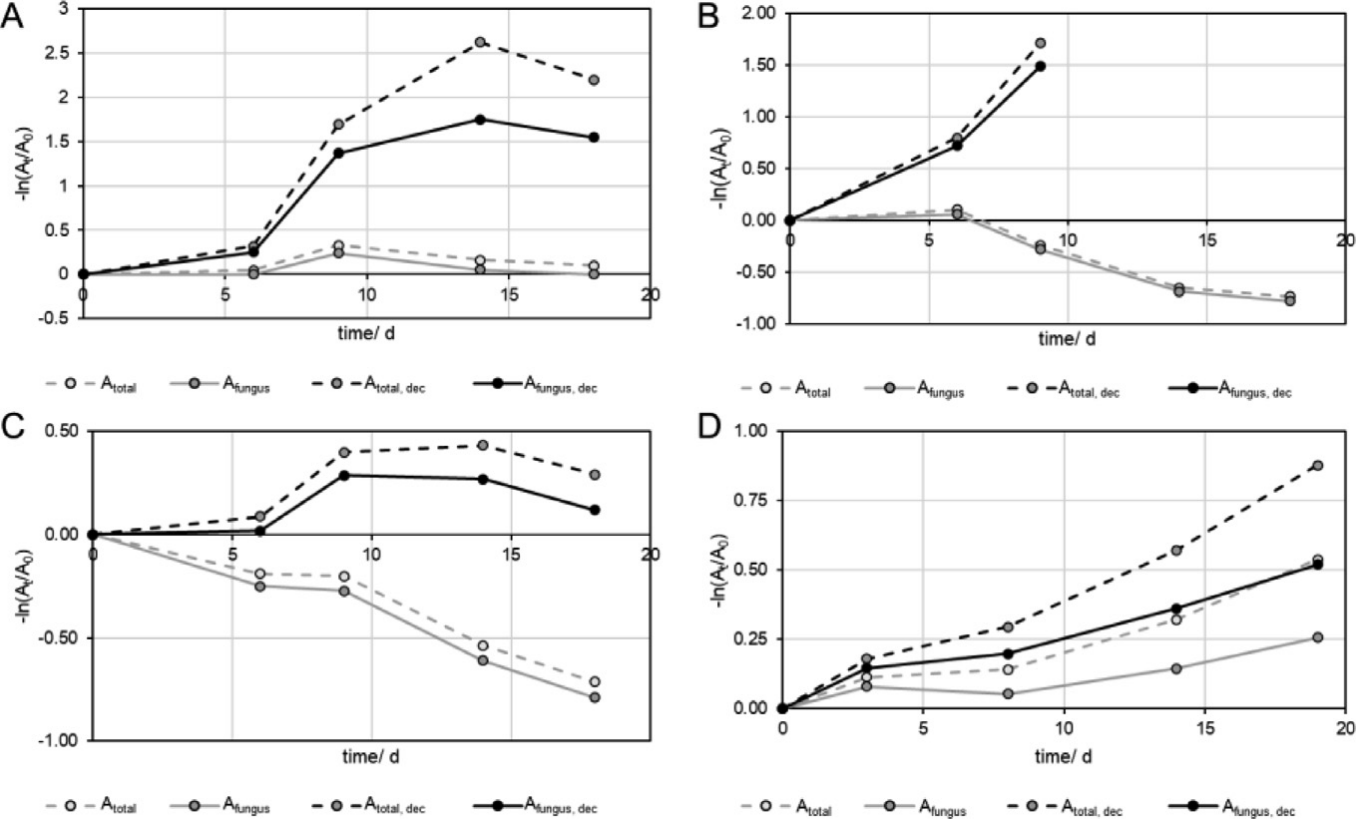
Linearized form (first order decay) of the absorption transients of measurements of solutions with dyes and with fungi (+D +F) obtained at different wavelengths with A) 597 nm for RB5, B) 595 nm for RB4, C) 630 nm for RG19, and D) 502 nm for RO16. The absorbance values were subtracted from the absorbance of the background (A_total_; grey dashed), from the samples that contained only dye and no fungus (A_fungus_), from A_total_ (A_total, dec_, black dashed), and from A_total,dec_ (A_fungus, dec_, black solid), at each sampling point. The absorbance is shown as mean of quadruplicates.

Decolorization assays are composed of samples containing the dye and the fungus of interest. The simplest calculation of the decolorization is made by subtracting the background absorption of the growth medium (A_total_) at each sampling point (Fig. 4 grey dashed). However, this simple calculation results in different shapes of the transients. The decolorization of RB5 showed a reduction of the absorbance until day 9 followed by an increase (Fig. 4 A grey dashed), RB4 showed an almost constant absorption and an increase for the later sampling points (Fig. 4 B grey dashed), as well as RG19, which remained almost constant until day 9 and increased later (Fig. 4 C grey dashed). A decrease from the first sampling point was only observed in the case of RO16 (Fig. 4 D grey dashed). Adding the proportion of dye that was decolorized in the absence of fungus (A_fungus_), returned less loss of absorption, as the contribution of the non-enzymatic degradation of the dye was added. As all dyes degraded slowly, but linearly the contribution of the dyes added to higher absorbance with later sampling points (Fig. 4 D grey solid). However, these data still contained the previously described absorption increase from the metabolites of the fungus. The contribution of this additional absorption can be removed by subtracting the data from the experiments with fungus and without dye (-D+F) from the results of the fungus with dye (+D+F), which resulted in a reduction of the absorbance (A_total, dec_), that increased with sampling points, as the absorbance from the metabolites of the fungi was increasing at later sampling points, and more dye was degraded at the same time (Fig. 4, black dashed lines). This calculation considers the contribution of the non-enzymatic and the enzymatic degradation and describes the overall decolorization of each dye (A_total, dec_). The calculations show that RB5, and RB4 were degraded faster than RO16, which could not be observed before, as the increasing absorbance of the metabolites were stronger at 597 and 595 nm, compared to the absorbance loss by decolorization. In case of RO16, a stronger decolorization was found than initially expected, and for RG19 almost no decolorization could be found at all. To obtain only the enzymatic decolorization efficiency, the contribution of the non-enzymatic degradation had to be removed from the absorbance data by adding the absorbance that was reduced during that process (Fig. 4, black solid). This addition increased the absorbance in cases of RB5, RB4, and RG19 (Fig. 4, A-C black solid) only slightly, but more in the case of RO16 (Fig. 4, D black solid lines), as the non-enzymatic degradation was larger in the latter case. The data that was obtained for the different calculations can be converted to rate constants (Eq. 2) and half-lives (Eq. 3) by simple first order decay models. Fig. 5 shows the linearized absorption data for the first order kinetic models, where the slopes indicate the rate constants (k), which are given in Table 3. Reasonable kinetic parameters for all types of calculations were only found for RO16 (Fig. 5 D), as the influence of the absorbance increase from the metabolites of the fungus was lower than for all other dyes. Although, a total half-life of 23.6 d (A_total_) was found for RO16, the calculations allowed to determine that the overall (enzymatic and non-enzymatic) degradation had a much shorter half-life of 14.3 d (A_total, dec_), and that the enzymatic decolorization had a half-life of 24.4 d (A_fungus, dec_). In this case, the absorption increase caused by the metabolites compensated the for the non-enzymatic degradation. In cases of RB4, and RG19 (Fig. 5 B, C), the data for A_total_ (grey dashed) and A_fungus_ (grey, solid) were dominated by the absorbance increase of the metabolites, which is indicated by the negative slopes, and respectively negative half-lives. RB5 (Fig. 5 A) shows an almost horizontal slope for A_total_ (grey dashed) and A_fungus_ (grey, solid), as the absorbance increase by the metabolites of the fungi and the decolorization was balanced. After subtraction of the absorbance that was contributed by the metabolites of the fungi, positive slopes are found (A_total, dec_) for RB5, RB4 and RG19 that indicate the decolorization of the dyes (Fig. 5 A-C, black solid and dashed lines). In case of RB4 (Fig. 5 B), the absorbance reached negative values and could not be calculated for 9, 14, and 18 days, indicating, that the RB4 was entirely decolorized before day 9. After calculation of the overall decolorization (A_total, dec_) the fastest degradation was found for RB4 (τ_1/2_=3.8 d^−1^), followed by RB5 (τ_1/2_=4.6 d^−1^), and RO16 (τ_1/2_=14.3 d^−1^), with the lowest rate for RG19 (τ_1/2_=33.5 d^−1^). The pure enzymatic degradation (A_fungus, dec_) dominated the overall degradation of RB5 (τ_1/2_=6.8 d^−1^), and RB4 (τ_1/2_=5.8 d^−1^), and to a minor extent RO16 (τ_1/2_=24.4 d^−1^), while it had a subordinate role in the degradation of RG19 (τ_1/2_=64.7 d^−1^).

(2) $$-ln\frac{A_{t}}{A_{0}}=kt$$

**Table 1:** Volumes and reagents added to each experiment.

	Growth medium/ µl	dye stock/ µl	fungus stock/ µl	Water/ µl	Sum/ µl
-D -F	480	0	0	20	500
+D -F	380	100	0	20	500
-D +F	480	0	20	0	500
+D +F	380	100	20	0	500

**Table 2:** Description and calculation of different absorbance data.

Calculation	Description
A_total_ = A_(+D+F)_ - A_(-D-F)_	A_total_ is the absorbance of the experiments containing fungi and dyes from which the background absorption of the growth medium is subtracted.
A_fungus_ = A_total_ + ΔA_(+D-F)_	A_fungus_ is the absorbance of the experiment containing fungus and dye to which the contribution of the non-enzymatic degradation of the dye is removed. Therefore, A_fungus_ represents the absorbance that is contributed by any fungus related process on the dye.
A_total, dec_ = A_(+D+F)_ - A_(-D+F)_	A_total, dec_ is the subtraction of the absorbance of the experiment containing fungus and dye from the absorbance obtained from the experiment containing fungi, but without dyes. Therefore, the resulting absorbance change is caused by any enzymatic and non-enzymatic processes on the dye and represents the overall decolorization
A_fungus, dec_ = A_total, dec_ + ΔA_(+D-F)_	A_fungus, dec_ is the absorbance obtained from the addition of the absorbance from the experiment without fungus and with dye to A_total, dec_. This calculation removes the contribution of the non-enzymatic degradation of the dye and results in the absorbance changes due to the pure enzymatic degradation by the fungi.

**Table 3:** Kinetic parameters of first order decay models of RB5, RB4, RG19, and RO16.

Dye	Absorbance	k/ d^−1^	R²	τ_1/2_ / d
RB5	A_total_	0.0068	0.1373	101.5
	A_fungus_	0.0008	0.0025	921.3
	A_total, dec_	0.1497	0.8183	4.6
	A_fungus, dec_	0.1022	0.7979	6.8
RB4	A_total_	-0.0492	0.8272	-14.1
	A_fungus_	-0.0511	0.8571	-13.6
	A_total, dec_	0.1820	0.9477	3.8
	A_fungus, dec_	0.1197	1.0000	5.8
RG19	A_total_	-0.0403	0.9493	-17.2
	A_fungus_	-0.0442	0.9718	-15.7
	A_total, dec_	0.0207	0.5721	33.5
	A_fungus, dec_	0.0107	0.3089	64.7
RO16	A_total_	0.0290	0.9265	23.9
	A_fungus_	0.0132	0.8777	52.4
	A_total, dec_	0.0484	0.9604	14.3
	A_fungus, dec_	0.0284	0.9820	24.4

Eq. 2: Linearized first order decay function used for calculating the rate constants of decolorization reactions, with A_t_ being absorbance at a given sampling point, A_0_, being initial absorbance, k being the rate constant, and t time (in days).

(3) $$\tau_{1/2}=\frac{\text{ln}\left(2\right)}{k}$$

Eq. 3: Calculation of the half-life (τ_1/2_) form first order decay functions, with the rate constant k.

## CRediT authorship contribution statement

**Christian Zafiu:** Writing – review & editing, Conceptualization, Data curation, Writing – original draft, Visualization, Methodology, Formal analysis. **Seta Küpcü:** Writing – review & editing. **Mika A. Kähkönen:** Writing – original draft, Investigation, Supervision, Writing – review & editing, Conceptualization, Methodology, Resources.

## Declaration of Competing Interests

The authors declare that they have no known competing financial interests or personal relationships that could have appeared to influence the work reported in this paper.

## Data Availability

Data will be made available on request.

## Additional information

### Background information to white rot fungi and their use in bioremediation

White rot fungi are microorganisms that degrade aromatic lignocellulose effectively, by secreting ligninolytic oxidative enzymes [1]. The variety of enzymes, which is produced by such fungi is ranging from cellulose- to lignin-degrading enzymes, which can degrade woody plants completely. In particular, non-specific ligninolytic enzymes, which include laccases, manganese peroxidases, and lignin peroxidases have the potential to degrade a wide range of xenobiotic compounds, such as aromatic amines, phenolic compounds, aliphatic polyalcohols, and polycyclic aromatic hydrocarbons (PAHs) [2], [3], [4], [5]. Therefore, the capability of white rot fungi and their enzymes for bioremediation was explored intensively, particularly for decolorizing textile dyes in effluent waste water of the textile industry [6,7] and for the degradation of cytostatic drugs [8]. The research of the past years focused on biotechnologically produced and purified fungal enzymes, which were already widely used, as they exhibit fast turn-over rates. [9], [10], [11], [12]. The direct enzymatic treatment allows a more controlled degradation when the activity of the involved enzymes is known. However, in an environmental setting, the enzymatic activities can be altered by their deactivation due to pollutants, such as heavy metal ions [9], which are often found as co-pollution e.g. in waste water from the textile industry [13,14]. Such a deactivation of the fungal enzymes also inhibits the remediation process. Usually, these enzymes cannot not be recovered, which makes an addition of more enzymes necessary to maintain the remediation process. In a pure bioremediation process, the living microorganisms produce enzymes continuously and perform similarly as pure enzymes on the decolorization of textile dyes in the presence of co-pollutants [15]. Moreover, in the pure biological treatment a broader variety of enzymes is produced, which could be advantageous towards mixtures of co-polluting xenobiotics. In addition, to the extracellular enzymes, white rot fungi exhibit also intracellular enzymes, such as cytochrome P450, which has also been shown to degrade persistent organic molecules, such as pesticides, effectively [16], [17], [18]. However, the isolation of cytochrome P450 and maintaining its activity after extraction is difficult and therefore currently not applicable to biotechnological processes [5]. Therefore, these enzymes are only available in bioremediation approaches that use living fungi.

*P. velutina* is a well-studied white rot fungus, that can be grown on the laboratory scale and was already investigated for its potential to be used for bioremediation of e.g. sawmills [19]. This type of fungus and its enzymes was already shown to degrade a variety of persistent chemicals such as the persistent organic pollutants (POP) polychlorinated dibenzo-p-dioxins (PCDD) and dibenzofurans (PCDF) [19], poly aromatic hydrocarbons (PAH) [20], 2,4,6-trinitrotoluene (TNT) [21], as well as the reactive textile dye Reactive Orange 16 [15], at high efficiencies. Among other white rot fungi, *P. velutina* represents a promising candidate for bioremediation purposes and a proper microorganism to study and benchmark degradation processes of persistent chemicals.

### General information on reactive dyes

Reactive dyes are chromophores that can undergo covalent bonds with textile fibres such as wool, cotton and polyamide. Due to their strong binding towards the fibres, these chemicals became popular as they maintain a strong and long colouring of products [22]. Among these reactive dyes for Reactive Orange16 (RO16), Reactive Black 5 (RB5), Reactive Blue 4 (RB4), and Reactive Green 19 (RG19) represent commercially available and industrially often used dyes. Since these dyes are vastly used in industry, usually in countries with poor waste-water management, they became an environmental problem. The dyes exhibit two unfavourable properties with one being the strong absorption of light that inhibits photosynthesis of plants and phytoplankton in water bodies and second, due to still remaining reactivity of some dyes that were excessively used and washed out [23]. Both properties lead to a reduction of oxygen in rivers and can cause immense environmental damages.

### Prospective applications of decolorization assays

Decolorization assays are simple methods to determine the potential of white rot fungi to degrade persistent coloured compounds, such as textile dyes. However, using living white rot fungi introduces the challenge that the microorganisms produce metabolites, that can interfere with the assay at wavelengths that are used also to follow the decolorization of the dye of interest. In certain cases, the interference introduces stronger absorption over time than the initial absorbance of the dye and can lead to the wrong conclusion that these dyes are not degraded. Using appropriate control experiments, that allow to assess the extent of the interference and subtracting the additional absorbance, allows to elucidate the decolorization performance. Moreover, using additional controls that allow to determine the contribution of the non-enzymatic decolorization of the dye allow to determine kinetic parameters that can be used to assess the efficiency of the fungal enzymes towards degrading the dye separately. Such assays can be usefully also for screening white rot fungi for potent enzymes that could be isolated and produced for bioremediation purposes.
